# Supplementary material for: Communicating social responsibilities through CSR reports: Comparative study of top European and Asia-Pacific airlines
Source: PLoS One. 2021 Oct 14;16(10):e0258687. doi: 10.1371/journal.pone.0258687 (PMC8516251; doi:10.1371/journal.pone.0258687)
Supplement: Attachment — Submitted filename: Response to Reviewers.pdf [file pone.0258687.s003.pdf]

Dear László Vas and reviewers:

Thank you for your letter and the reviewers' comments on my manuscript entitled " Communicating social responsibilities through CSR reports: Comparative study of top European and Asia-Pacific Airlines " (ID: PONE-D-21-20662). Those comments are very helpful for revising and improving my paper, as well as the important guiding significance to my future research. I have studied the comments carefully and made corrections which I hope meet with approval. In the revised manuscript, the supplementary parts were all highlighted within the document by using red-colored text, and the responds to the reviewers' comments are as follows.

Replies to the reviewers' comments:

Reviewer #1:

1. "Since the topic of CSR is relevant and actual, the literature should focus on more resent resources as well."

Response: Thank you for your advice. I took a thorough check to my paper and add citation of recent studies in the Introduction [3,4], the Literature Review [23,24] and the Discussion and Conclusions [54,57].

2. "The sampling is not described in detail. How were the 10-10 companies selected from the list? Random sampling?"

Response: Thank you for your advice on the sampling part. I have added explanation of sampling method in the revised version (Page 4, Line 126-138).

Reviewer #2:

I really appreciate your recognition and encouragement to my study.

Once again, thank you very much for your constructive comments and suggestions which would help me in depth to improve the quality of the paper.

Your sincerely,

Xing Zhang

Faculty of Business, City University of Macau
School of Management, Guizhou University of Commerce, China

Email: B19092100063@cityu.mo
